# Effects of different fatigue locations on upper body kinematics and inter-joint coordination in a repetitive pointing task

**DOI:** 10.1371/journal.pone.0227247

**Published:** 2019-12-31

**Authors:** Chen Yang, Samuel Leitkam, Julie N. Côté

**Affiliations:** 1 Department of Kinesiology and Physical Education, McGill University, Montreal, Quebec, Canada; 2 Occupational Biomechanics and Ergonomics Laboratory, Michael Feil and Ted Oberfeld/CRIR Research Centre, Jewish Rehabilitation Hospital, Laval, Quebec, Canada; North Carolina State University, UNITED STATES

## Abstract

Studies have shown that muscle fatigue can lead to posture, joint angle, inter-joint coordination and variability alterations. However, the three-dimensional kinematic effects of localized muscular fatigue on a multijoint movement remain unclear. Healthy young adults (N = 17, 10 females) performed a standing repetitive pointing task when they were non-fatigued, and after localized muscle fatigue was induced at the elbow, the shoulder, and the trunk using isometric protocols performed until exhaustion. Joint angles and angular standard deviation (SD) of trunk, shoulder and elbow, and continuous relative phase (CRP) and CRP SD between trunk and shoulder, and shoulder and elbow were computed and compared between fatigue conditions. Results showed that trunk lateral flexion SD increased after fatigue of the elbow (0.1°, p = 0.04), shoulder (0.1°, p = 0.04) and trunk (0.1°, p<0.01). However, fatigue at different muscles brought different kinematic changes. Shoulder fatigue induced the greatest overall changes, with angular changes at all three joints. Trunk fatigue increased the shoulder horizontal abduction SD, elbow flexion SD and trunk-shoulder CRP. Elbow fatigue induced angular changes at trunk, shoulder and elbow, but did not affect CRP or CRP SD. This study highlights the crucial role of trunk variability in compensating for localized muscle fatigue during a repetitive upper limb task performed while standing.

## Introduction

Repetitive upper limb movements are regularly performed in varieties of jobs as well as in the activities of daily living. However, sustained repetitive movement is associated with musculoskeletal disorders (MSDs) [1, 2]. MSDs of the upper limb and trunk accounted for 53% of the 240,682 lost time claims in Canada in 2016 [3]. Hence, the huge economic burden that MSDs have brought to our society cannot be ignored. Muscular fatigue is one of the most important factors contributing to the development of MSDs [4]. Thus, a better understanding of the mechanisms of muscular fatigue would help us better understand the mechanism of MSDs, which could help their prevention.

Muscular fatigue can be defined as a time-dependent process illustrated by increased muscle activity amplitude and spectral shift to lower frequencies in the fatigued musculature at the given force level [5]. The effects of muscular fatigue have been well described in terms of muscle force, power output, electromyogram (EMG) characteristics as well as body posture and kinematics [6–9]. Posture and movement adaptations to fatigue have been interpreted as compensatory strategies to reduce the load on the fatigued musculature [9]. Previous studies on shoulder fatigue indicated trunk movement changes in a repetitive pointing task [9, 10]. In addition, Davidson et al. [11] induced lumbar extensor fatigue and found increases in whole-body centre of mass (CoM) and centre of pressure (CoP) displacements during a postural task. They proposed that lumbar extensor fatigue would lead to a proprioception deficit, thus increase in instability and in angular movements at the lumbar joints as well as in the CoM and CoP displacements. However, none of the studies have examined the effects of trunk fatigue on posture and upper limb kinematic parameters in a multi-joint upper limb task.

Other studies have quantified coordination, i.e. the relationship between two biomechanical parameters implicated in movement control, to study how multijoint movement is affected by fatigue. Coordination, which represents the nervous system’s process to master the redundant degrees of freedom to produce a controllable system, is crucial to generate a desired movement [12]. As such, inter-joint coordination adaptations to muscle fatigue have been highlighted in various studies [13–15]. Côté et al. [13] found that even though repetitive motion-induced fatigue led to decreases in elbow motion amplitude, some other joints increased their motion amplitudes, resulting in an ability to maintain the endpoint trajectory in a sawing task. Forestier and Nougier [14] found an absence of temporal delay between the elbow and hand peak velocity after elbow extensor fatigue in a ball-throwing task, suggesting a more rigid movement organization to simplify movement execution and control.

Continuous relative phase (CRP) is a method that measures the phase angle difference between two segments, and can provide a quantitative assessment of both spatial and temporal aspects of coordination between two linked joints [16]. Moreover, variability (e.g. standard deviation) of the coordination is believed to reveal flexibility of the movement pattern and of task execution [17]. Recently, we investigated the CRP and its variability across repeated movements, using CRP standard deviation, between shoulder and elbow motion in a prolonged pointing task [16, 18]. Results showed that with fatigue, coordination variability (CRP standard deviation) increased while the endpoint variability was maintained. However, most of these studies have induced muscle fatigue at one single joint or through repetitions of one multijoint task. In everyday tasks, muscle fatigue can be induced at different parts of the body, due to the diversity of tasks that workers perform on a daily basis at work.

To perform multi-joint tasks, various degrees of freedom need to be coordinated. However, when muscle fatigue is induced at different parts of the multi-joint linkage, it is plausible that the inter-joint coordination would change differently [19, 20]. According to the leading joint hypothesis, the role of the leading joint (the most proximal joint in an upper limb multijoint task) is to create the main acceleration/deceleration for the entire limb. As for the subordinate joints, their roles are to regulate the impact from the leading joint in order to produce a desired movement [21]. Only two studies examined the postural and coordination difference of proximal arm joint fatigue and distal joint fatigue. In 2005, Huffenus et al. studied the effects of proximal (triceps branchii) and distal (extensor digitorum communis) fatigue on multi-joint movement organization in a throwing task [20]. When the distal muscles were fatigued, the elbow active torque decreased and the wrist angular velocity was maintained. However, when the proximal muscles were fatigued, the wrist angular velocity was increased. Cowley and Gates [19] compared the effects of proximal (shoulder flexors) and distal (finger flexors) muscle fatigue on movement coordination in a wrenching task. After the proximal fatigue condition, trunk lean angle, angular velocity and elbow flexion angle increased while humeral elevation angle decreased. After the distal fatigue condition, the trunk lean angle and wrench relative to hand velocity increased, and the peak wrist extension was earlier.

In summary, the previous research has ignored how trunk fatigue can affect the performance of a standing upper limb repetitive task, nor has anyone investigated joint amplitudes or kinematic coordination during such a task as a function of localized joint fatigue. Thus, the purposes of this study were to assess the effects of fatiguing different body regions on the 3D kinematic characteristics (joint angles, angular variability, inter-joint coordination, coordination variability) in a repetitive upper limb task. We hypothesized that fatigue at different parts of the body would lead to different changes in terms of 3D joint angles during the performance of a repetitive upper limb task. Based on the leading joint hypothesis, the variability of the fatigued joint would increase the most with trunk fatigue and increase the least or stay the same with elbow fatigue.

## Methods

### Participants

Seventeen right-handed healthy young adults (7 men, 10 women; age = 23 ± 2.7 years; height = 172.9 ± 8.8 cm; body mass = 64 ± 10.2 kg) were recruited to participate in this study. The participants were recruited through the printed and electronic advertisements on notice boards (from 2017 June to August). All participants were university students from the McGill University campus. Participants were excluded if they had any previous experience in manual material handling (MMH) work, had any lower back pain, upper body injuries, musculoskeletal or cardiovascular impairment in the last 6 months before the data collection. The participants were believed to represent the young healthy adult right handed population with no MMH work experience. All participants provided written informed consent prior to participation. The individuals in the photos have given written informed consent to publish these case details. The study was approved by the Research Ethics Board of the Centre for Interdisciplinary Research in Rehabilitation (CRIR) of Greater Montreal, and conducted in accordance with The Helsinki Declaration.

### Experimental protocol

**Fig 1:**
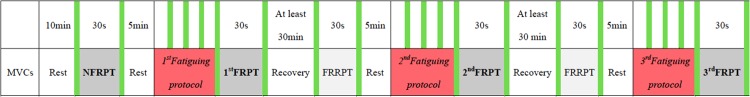
The flow chart of the data collection protocol. The series of shoulder, elbow and lower back fatiguing protocols were performed in random order. The green lines indicate when the Borg CR10 score was asked. MVCs, NFRPT, FRPT, FRRPT stand for maximal voluntary contractions, non-fatigued RPT, Fatigued RPT, Fatigue Recovered RPT, respectively.

A brief introduction of the data collection process was given after the participants signed the consent form. Then the participant performed a few familiarization trials of the repetitive pointing task (RPT). The RPT was performed as described in Fuller et al. [9] with few changes. Briefly, the participant repetitively moved his/her right arm between a proximal and a distal target aligned with their body midline at shoulder height while standing. In the current project, the rhythm was maintained at one forward and one backward movement per second (1 second for a full cycle). The touch-sensitive cylindrical targets provided auditory feedback to help the participant keep the pace. In addition, in the current project, participant held a weight (12cm * 7.5cm * 1cm, 0.7 kg for females, 1.4 kg for males) while performing the RPTs, in order to imitate an assembly line task of a real-life work environment. After the participant practiced performing the RPT as instructed, he/she had ten minutes to rest. After ten minutes, the participant performed the RPT for 30 seconds as the non-fatigued RPT (NFRPT). The Rated Perceived Exertion (RPE) of the shoulder, triceps branchii and lower back muscles were asked using the Borg CR-10 scale before and after the NFRPT [22]. Then, the participant performed a series of fatiguing tasks to fatigue the muscle of shoulder, elbow and trunk one by one. The order of the three fatiguing protocols was randomized, and right after each fatiguing protocol, the participant was asked to perform another 30 s RPT as the Fatigued RPT (SFRPT for shoulder fatigued RPT, EFRPT for elbow fatigued RPT, TFRPT for trunk fatigued RPT). In between fatiguing sequences, the participant was instructed to sit on a chair and passively recover for at least 30 min [11]. The Borg CR-10 exertion scale score of the target muscles was asked every 5 minutes, and the recovery period continued until the Borg exertion score of the target muscle went back to the same number as the one before the NFRPT. The whole protocol process is shown in Fig 1.

**Fig 2:**
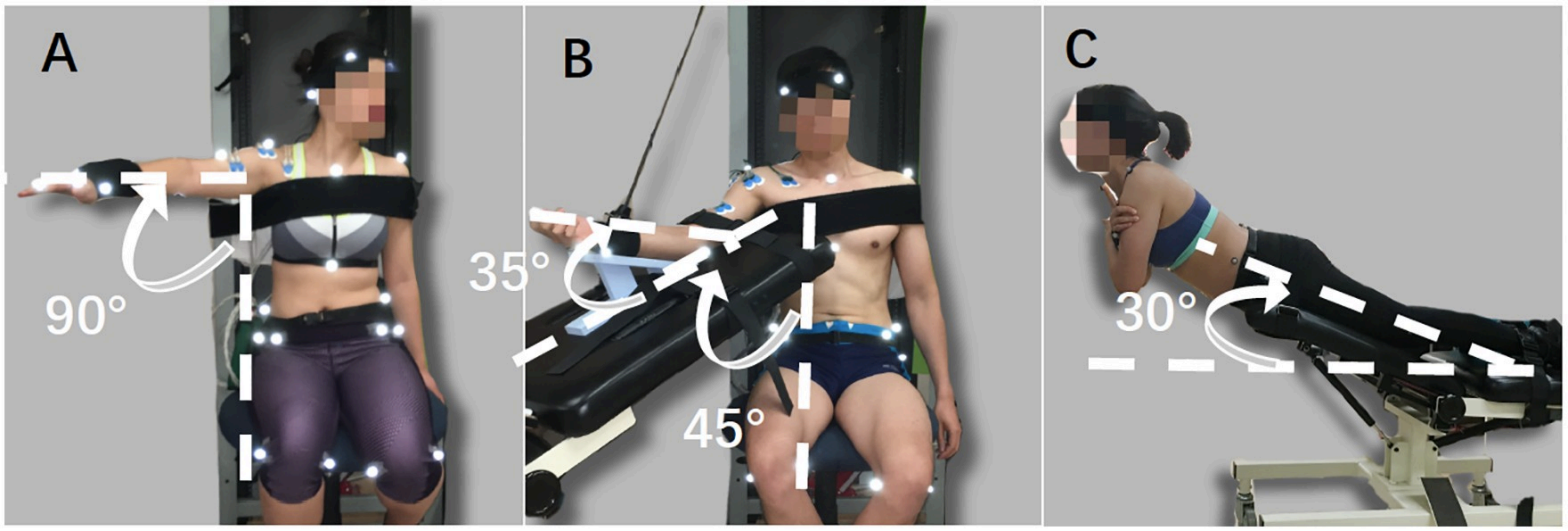
Shoulder, elbow and lower back fatiguing protocol setups. From the figure A to C are the figures showing the shoulder, elbow and lower back fatiguing protocols, respectively.

The fatiguing protocols for shoulder, elbow and lower back muscles were conducted in series of intermittent static tasks. Specifically, the shoulder fatiguing protocol was a series of intermittent static task designed to fatigue the muscular structure around the shoulder joint (Fig 2A). The participant was asked to sit on a chair against a metal frame behind the back. The chair height was adjusted to keep the knees at an angle of 90°, and the upper body of the participant was tied to the metal frame behind them by Velcro straps to stabilize and prevent the upper body from moving. While holding an extra weight (12 cm * 7.5 cm * 1 cm, 0.7 kg for females and 1.4 kg for males) inserted to the wrist band, he/she was to elevate their right arm to 90° of shoulder flexion and 45° of horizontal abduction. Then, the participant was asked to hold this position 1 minute per set once the fatiguing protocol began. Ten seconds of break was given between consecutive sets. The series of elbow fatiguing protocol was designed to fatigue the elbow extensors (triceps branchii). It was performed on the chair with the same instruction as the shoulder fatiguing protocol. The right arm was placed on a massage chair beside the participants (Fig 2B). The right wrist band was attached to a Thera-Band tube (black color, 3.31 kg of resistance at 100% elongation), and the other end of the Thera-Band tube was attached to the metal frame behind the participant. The participant was instructed to extend his/her elbow until the forearm touched the blue triangle frame on the massage chair during the fatiguing protocol. This posture was to be held for 1 minute per set, with 10 seconds between sets. Finally, the target muscles of the lower back fatiguing series were the erector spinae muscles. A massage bed was adjusted to 30° as shown in Fig 2C to play the role of a Roman chair. The hips, knees and ankles of the participant were tied to the chair using a strap. The participant was asked to cross his/her arms in front of the chest and elevate the upper body until it was aligned with the legs during the fatiguing protocol. He/she was to hold this posture for 30 seconds per set, and 10 seconds break was provided between sets. The Borg CR-10 scale [22] score for the target muscles was asked at the end of each fatiguing set. The stoppage criteria were either: (1) the participant reached Borg score 10 (out of 10) for three times in a row, or (2) the posture could not be maintained for 1 min (30 seconds for trunk) three times in a row. The participant was not aware of these stoppage criteria.

### Data acquisition

A 7 camera motion capture system (MX3 VICON, Oxford Metrics Ltd., Oxford, UK) was used to record kinematics (sampling frequency = 100 Hz). Reflective markers (12 mm) was placed on the trunk (C7, left and right T8, Incisura Jugularis and Xiphoid Process), upper arm (acromioclavicular joint, lateral and medial epicondyle), forearm (lateral and medial epicondyle, styloid processes of ulna and radius), hand (styloid processes of ulna and radius, second and fifth metacarpophalangeal joint, index fingertip), pelvis (left and right anterior superior iliac spine, greater trochanter and S1) [23, 24]. The RPE of shoulder, elbow and lower back muscles was rated by the participants before and after each RPT trial (NFRPT, FRPT, FRRPT) using the Borg CR-10 exertion scale [22]. The RPE of the target muscle was rated every 5 minutes during the recovery session (as shown in the Fig 1).

### Data analysis

Kinematic data was low-pass filtered (digital 2th order Butterworth filter, cut-off frequency = 7 Hz, zero phase lag) in Visual 3D (C Motion, Germantown, MA). A 6-segment model was created to calculate the trunk-pelvis, shoulder, and elbow angles. Shoulder and elbow angles were defined and calculated in Visual 3D as described in Gates et al. [25]. According to the ISB guideline, we recognized the shoulder angles as followed: the first rotation (Y’) as the plane of elevation; the second rotation (X) as the elevation; the third rotation (Y”), internal/external rotation as the axial rotation angle. Trunk-pelvis, shoulder and elbow kinematics were calculated using Euler angles according to International Society of Biomechanics recommendations [26]. The coordination between shoulder and elbow, shoulder and trunk was assessed using the continuous relative phase (CRP) as described in Yang et al. [18], Hamill et al. [27]. Specifically, the phase angle of one joint was calculated for any time point in one cycle as Hamill et al. [27]. Then, the CRP at each time point was calculated as the difference between the phase angles of the proximal joint and the distal joint (e.g. CRP between shoulder and elbow is the relative phase angle of shoulder minus the relative phase angle of elbow). As stated in Yang et al. [18], 0° means two joints are moving in phase and 180° means moving antiphase. The CRP of the following joint angles were calculated: shoulder plane of elevation and elbow flexion/extension, trunk flexion/extension and shoulder plane of elevation, trunk rotation and shoulder plane of elevation, trunk lateral flexion and shoulder plane of elevation, trunk lateral abduction/adduction and shoulder elevation. For each of those variables (joint angles and CRPs), data from each forward movement cycle was first time normalized to 101 data points. The average and SD values of the 101 points were calculated for each cycle. Afterwards, the mean value of all the complete movement cycles (i.e. excluding data from the first and last 5 cycles to avoid accounting for incomplete cycles, and to avoid data boundary issues, i.e. cycles when the participant was accelerating to get into the rhythm, or decelerating to prepare to stop) was calculated to get the mean joint angle or mean SD value. Likewise, the maximum and minimum values were also calculated to get the mean maximum and mean minimum value. We calculated the mean, maximum, minimum and SD joint angles to represent the joint angles and angular variability in all RPT trials. As for CRP, the mean CRP is a measure of inter-joint coordination while the CRP SD is our chosen metric for coordination variability.

### Statistical analysis

Generalized estimating equations (GEE) were used to examine the effects of fatigue location (NFRPT, SFRPT, EFRPT and TFRPT) on each kinematics variables (joint angles, angular variabilities, CRP and CRP variabilities). The GEE approach was selected because it has more power than repeated measures analyses of variance (RM-ANOVA), it is less restrictive in its assumptions than RM-ANOVA, it helps estimate the average change per group, it is robust against a misidentified choice of correlation matrix [28, 29]. The LSD tests were used to apply the paired-wise comparisons (NF vs. EF, SF and TF; EF vs. NF, SF and TF; SF vs. NF, EF and TF; TF vs. NF, EF and SF). Benjamini-Hochberg procedures were applied to correct the p values to avoid the type I error [30]. The false discovery rate was set at 5%. Statistics were performed in SPSS (SPSS Statistics v24, IBM Corp., US) and Excel (Microsoft^®^ Excel for Windows Version 15.26, Microsoft., US).

## Results

**Fig 3:**
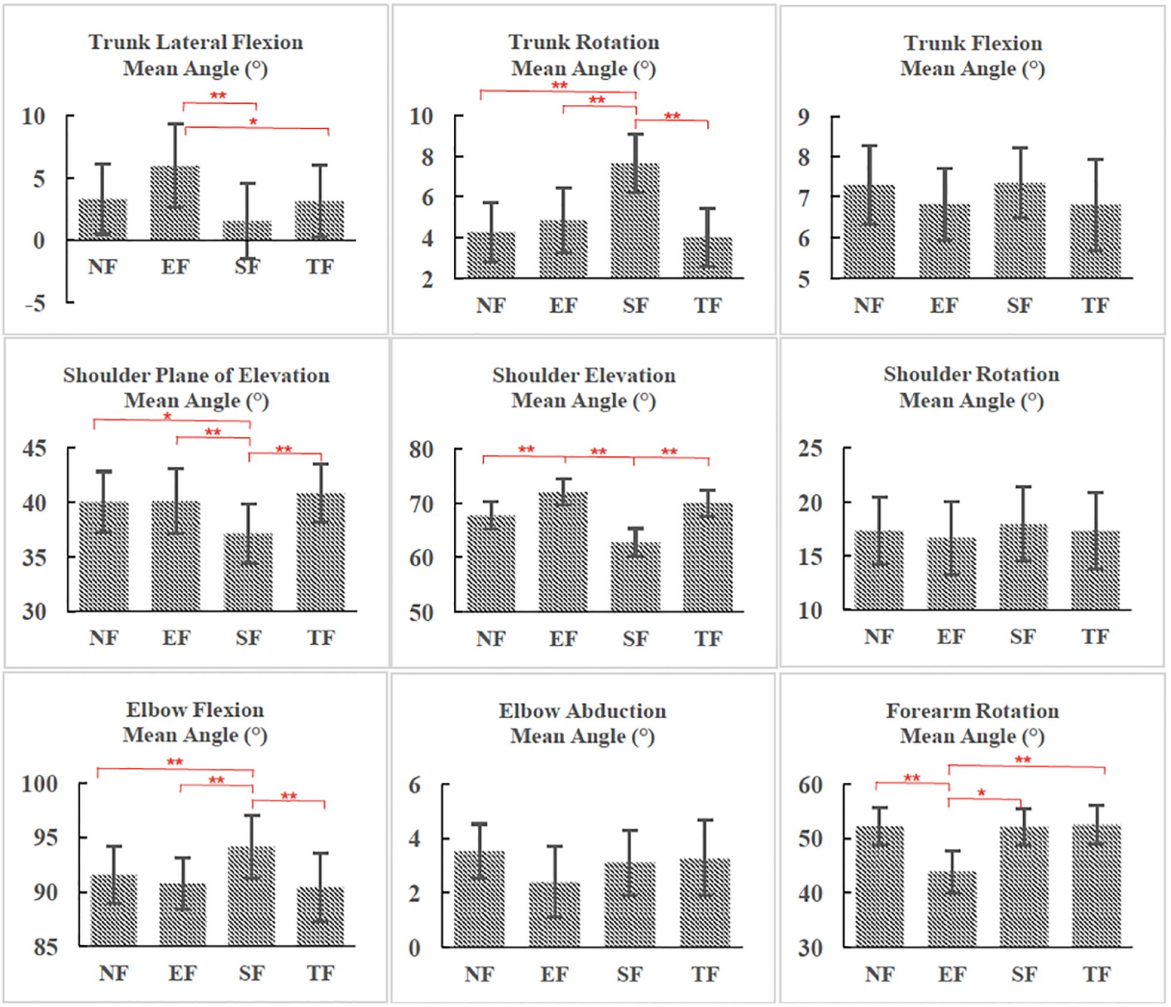
Joint angles between conditions (NF vs EF vs SF vs TF). *NF, EF, SF and TF represent non-fatigued RPT, elbow fatigued RPT, shoulder fatigued RPT and trunk fatigued RPT respectively. Angle definitions refer to the definitions by Gates et al. (2016) and We et al. (2005). The signs of trunk lateral flexion, trunk rotation, shoulder elevation, shoulder rotation and elbow abduction angles are changed to obtain the positive values. Positive values in trunk lateral flexion, trunk rotation and trunk flexion angle means bending towards the non-reaching side, rotating towards the reaching side and bending forward respectively; Positive shoulder elevation angle and shoulder rotation angle stands for humerus horizontal flexion forward, humerus elevation, humerus external rotation. Positive elbow flexion, elbow abduction and forearm rotation means forearm flexion, forearm abduction and pronation. * indicates p < 0.05, ** indicates p < 0.01.

**Fig 4:**
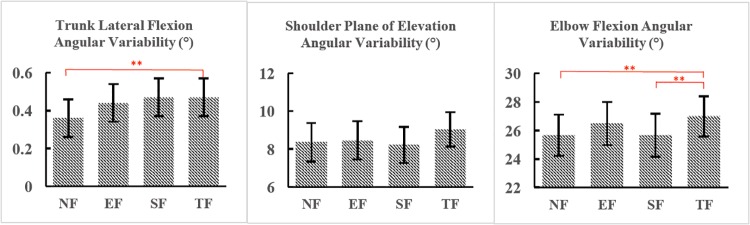
Joint angular variabilities between conditions (NF vs EF vs SF vs TF). *NF, EF, SF and TF represent non-fatigued RPT, elbow fatigued RPT, shoulder fatigued RPT and trunk fatigued RPT respectively. The letters after * above the bars indicate significant differences. * indicates p < 0.05, ** indicates p < 0.01.

On average, participants had performed 9.1±3.1, 8±1.9 and 8.9±3.2 trials for elbow, shoulder and trunk fatigue, respectively. Results show that different fatigue locations brought different kinematic effects during the performance of the repetitive pointing task (see Figs 3 and 4 and S1 Table). TF induced changes at elbow joint angle, trunk and elbow joint angular variabilities and trunk-shoulder CRP. SF had the greatest impact on whole-body kinematics, with changes in all joint angles, in trunk angular variability and in shoulder-elbow CRP variability. Finally, EF changed only the trunk and shoulder joint angles.

As shown in Fig 3, SF induced changes in mean angles at all three joints, as well as in the shoulder-elbow coordination variability. Specifically, the trunk lateral flexion mean angle was smaller after shoulder fatigue than it was after EF (4.4° smaller than after EF, p<0.01) and also the trunk mean rotation angle was greater after SF than any other conditions (3.4° greater than after NF, p<0.01; 2.8° greater than after EF, p<0.01; and 3.6° greater than after TF, p<0.01). These changes implied that the trunk was leaning less but rotated more towards the non-moving arm’s side after SF. As for the shoulder, the mean plane of elevation angle (2.9° smaller than after NF, p = 0.03; 3° smaller than after EF, p<0.01; and 3.71° smaller than after TF, p<0.01) and shoulder elevation angle (5° smaller than after NF, p<0.01; 9.3° smaller than after EF, p<0.01; and 7.3° smaller than after TF, p<0.01) were the smallest, meaning that the humerus was less forward and less elevated after SF, compared with all other conditions. The mean elbow flexion angle was also the greatest after SF (2.6° greater than after NF, p<0.01; 3.34° greater than after EF, p<0.01; and 3.7° greater than after TF, p<0.01), indicating that the elbow was on average more flexed. Besides, the minimum elbow flexion angle was greater after SF than it was after EF (3.5° greater, p<0.01) and TF (5° greater, p<0.01, see S2 Table). As for joint angular variabilities (Fig 4), SF brought changes at the trunk, where trunk rotation variability was 0.1° and 0.2° greater than it was after EF (p<0.01) and after TF (p<0.01), respectively. The greater trunk angular variabilities implied a more unstable trunk movement pattern after shoulder fatigue.

TF only induced joint angle changes to the elbow. The minimum elbow flexion angle (S2 Table) was smaller than it was after NF (2.6° smaller, p<0.01) and SF (5° smaller, p<0.01). The major changes that TF brought were in the joint angular variabilities and CRPs (see Fig 4 and S1 Table). The variability of elbow flexion was greater in TF than after SF and NF (1.4° greater than after NF, p<0.01; and 1.4° greater than after SF, p<0.01) (Fig 4). The greater variabilities at elbow suggested that fatigue at trunk could affect the movement patterns of the distal joints (elbow) at the multi-joint linkage. As for CRPs (S1 Table), the trunk rotation- shoulder plane of elevation CRP was greater after TF than after NF (3.7° greater than after NF, p = 0.03) and EF (5.6° greater than after SF, p<0.01), and finally, the trunk lateral flexion—shoulder elevation CRP was greater than after SF (by 11.4°, p = 0.03).

Lastly, comparing with all other conditions, EF only brought changes to mean joint angles, and none to joint angular variabilities, CRPs and CRP variabilities. However, the mean angle changes occurred at all three joints (Fig 3). The trunk mean lateral flexion angle was the greatest after EF compared to SF and TF (4.4° greater than after SF, p<0.01; and 2.8° greater than after TF, p = 0.03), meaning that after EF, the trunk was more flexed towards the non-moving arm’s side. The same occurred with the mean shoulder elevation angle (4.3° greater than after NF, p<0.01; 9.3° greater than after SF, p<0.01; and 2° greater than after TF, p<0.01), indicating that after EF, the shoulder was more abducted. Finally, the mean forearm rotation angle was the smallest in EF compared to in all other conditions (8.4° smaller than after NF, p<0.01; 8.2° smaller than after SF, p = 0.02; and 8.6° smaller than after TF, p<0.01), indicating less rotation after EF.

## Discussion

### Non-fatigue

Only one variable, trunk lateral flexion angular variability, was found to increase in all fatigue conditions, compared to NF (Fig 4), highlighting the important role of the trunk in this multi-joint movement. Muscle fatigue has been linked to increased motor variability in previous studies [31–33], angular variability being one way of measuring motor variability which can be defined as the natural variation in motor behavior. This has been found in Cowley and Gates [34] showing that both distal and proximal fatigue increased movement variability at the proximal joint (shoulder). Indeed, since the trunk is the most proximal segment and is the closest to the body Centre of Mass (CoM), this result can be perceived as a sign of general fatigue having a greater demand on global postural control in order to keep the CoM within the base of support. However, another way to interpret this result is that it may reflect a mechanically advantageous search for new movement strategies. The system may activate more degrees of freedom to compensate for the muscle fatigue to continue the task. In addition, lateral flexion occurs orthogonal to the main movement direction of the RPT. Thus, the system may choose to increase its variability in a direction that will have little impact on the overall objective of the task, which is to move the finger forward and backward, but that will help develop other compensatory strategies in all dimensions across the different joints of the kinematic chain. Similar findings were previously shown in Fuller et al. [35], where whole-body fatigue compensations were found mainly in directions orthogonal to the finger motion, and were interpreted as following a principle of minimal interaction between fatigue responses and task objectives.

### Shoulder fatigue

Kinematic compensation to shoulder fatigue has been well demonstrated in previous studies [13, 14, 36]. For instance, Fuller et al. examined the three-dimensional marker coordinates changes and joint coordinates variabilities with shoulder fatigue in RPT [9, 31]. However, our study is the first to provide evidence for the three dimensional joint angle and angular variability changes in RPT. In addition, the Fuller et al. studies fatigued the shoulder through repetition of the repetitive pointing task, whereas in the current study, was used static efforts to induce localised shoulder muscle fatigue (as well as elbow and trunk fatigue). When comparing shoulder fatigue with other fatigue locations (TF and EF), we observe that shoulder fatigue brought the greatest overall kinematic changes to the RPT movement pattern. SF changed the angles of all three joints (two planes for trunk and shoulder), increased the angular variability (two planes for trunk) as well as coordination variability. The smaller shoulder plane of elevation and elevation angles indicated that the shoulder was less forward and less elevated. These are believed to be signs of fatigue of one of the main shoulder agonists (deltoid). Similar findings can be found in Fuller et al. [9], where authors observed a less abducted and flexed shoulder angle in RPT after shoulder fatigue. Besides, Fuller et al. [9] also detected that the trunk was leaning towards the non-reaching arm’s side since the right shoulder marker was shifting higher and left. However, in our study, the trunk was bending less but rotating more towards the non-reaching arm’s side. The participants tended to bring the reaching shoulder closer to the targets by rotating the trunk more. This difference can be a result of the different fatiguing protocols combined with the faster arm movement speed in our study. Besides, the elbow flexion angle was greater in our study. The combination of greater elbow flexion angle and smaller shoulder plane of elevation angle revealed a decreased movement range at shoulder and elbow in the horizontal plane. However, the decreased movement range at shoulder and elbow was compensated by the increased contributions of trunk rotation. Similar multijoint compensatory patterns have been shown in previous studies where authors observed an increased range of motion at trunk when the arm was fatigued [13, 19].

In addition, SF brought greater variabilities of trunk rotation angle (Fig 4). Increased variability with fatigue has been previously well documented [23, 31, 33]. Even though the shoulder—elbow CRP variability was found to increase after shoulder fatigue in a previous paper [18], our results did not show the difference between SF and NF. This slight difference can be a result of differences in fatiguing protocols between the two studies.

### Trunk fatigue

Compared with all other fatigue locations, the only joint angle change that arose when fatigue was induced at the trunk was the elbow flexion/extension angle. The minimum elbow flexion angle (S2 Table) was smaller than SF and NF in TF, which indicated that the elbow was more extended when touching the distal target, possibly indicating a need to compensate for increased trunk rigidity with fatigue when the arm was extended towards the distal target. The most interesting results of TF were the variabilities (Fig 4). The variabilities of elbow flexion angle were greater than in SF and NF. These results imply that fatigue at the proximal joint (trunk) would increase the variability at distal joint (elbow). As supported by previous results, the increased variability at distal joints is believed to be a consequence of the fatigued proximal joint [18]. As proposed in the hierarchical control hypothesis and the leading joint hypothesis, muscles around one joint (identified as the leading joint) generate the movement of the whole linkage and in turn, the muscles around the neighboring joints produce corrections of the movement to fulfil the task [21, 37]. Our results show that the elbow produces the corrections (by the increased variabilities) of the movement to continue the task when the trunk is fatigued. This highlight the importance of hierarchical control hypothesis and is in line with the leading joint hypothesis.

Previous studies have discovered that muscle fatigue induces changes in the coordination between joints [14, 34, 38]. However, most studies focused on shoulder or upper limb muscle fatigue. Trunk as the most proximal segment/joint in the linkage is believed to affect distal joint movement variability as well as the inter-joint coordination. In this study, trunk—shoulder CRP changes were observed only in TF (S1 Table). This is in line with our hypothesis that trunk fatigue would change the coordination between joints, more so than the elbow fatigue. TF resulted in greater trunk—shoulder CRP, which means that after trunk muscles became fatigued, shoulder movement preceded trunk movement, implying that trunk fatigue delays trunk movement and needs to be compensated by earlier movement at the shoulder. However, Cowley and Gates [34] found that only distal fatigue caused changes in the temporal sequence of the joints, which is different from our result. Nonetheless, it was unknown if the proximal or distal fatigue affected the inter-joint coordination between two main contributors (e.g. shoulder—elbow or elbow—wrist). In our study, we used CRP as the inter-joint coordination variable, which allows a more advanced analysis of spatial and temporal coupling. The study by Huffenus et al. [20] showed that when the proximal joint was fatigued, the compensatory strategy used by the nervous system was to modify the multi-joint coordination. This supports our results and the interpretation of coordination adaptation to proximal muscle fatigue.

### Elbow fatigue

EF led to greater trunk lateral flexion angle, greater shoulder elevation angle and smaller forearm rotation angles than in all other conditions (Fig 3). This implies that the trunk was bending towards the non-reaching arm’s side, shoulder elevated higher and forearm pronated less. Very few studies examined the effects of distal muscle fatigue on multijoint coordination. Cowley and Gates also detected that the trunk would lean towards the not-moving arm’s side in a standing wrenching task when the distal (hand) muscle was fatigued [19]. However, they found that the trunk leaning angle was greater when the proximal (shoulder) muscle was fatigued. Our results showed that distal (elbow) muscle fatigue brought joint angle changes at trunk, shoulder and elbow. However, there were no CRP, angular variability (except for the trunk lateral flexion variability) or CRP variability changes when the elbow muscle was fatigued. These results suggest that elbow fatigue would not change the inter-joint coordination pattern, but rather, that people adapt to fatigue using a series of kinematic changes at all the joints in the linkage. This is also supported by the leading joint hypothesis, suggesting that the subordinate joint regulates the impact of the leading joint to generate the requested movement [39]. Hence, when the leading joint is fatigued, the subordinate joint movement would be modified to maintain performance (as shown in the results of TF), yet when the subordinate joint is fatigued, the initial multi-joint movement pattern may not be revised. However, our results only partly support this, and more experimental data is needed to further support theories of motor control such as the leading joint hypothesis.

### Limitations

The results of our study must be interpreted in light of some methodological limitations, such as small sample size, and the fact that we did not account for additional ranges of motion at other joints such as the knees and ankles. Besides, previous studies have shown sex differences in kinematic adaptations to muscle fatigue in the RPT [40], however, small sample sizes precluded us from performing these analyses here. Further research on sex differences is needed to better understand the impact of localized muscle fatigue locations.

## Conclusion

This study shows that localized muscle fatigue at either trunk, shoulder or elbow increases trunk lateral flexion variability in the repetitive pointing task. However, aside from this, fatigue at different locations has different impacts on joint angles, angular variability, upper body inter-joint coordination and coordination variability. This is the first study comparing the effects of localized muscle fatigue on angular kinematic characteristics across multiple trunk and arm joints during the performance of a standing repetitive upper limb task. Results can help understand how the body adapts to fatigue in various sport and work contexts.

## Supporting information

S1 Table Mean CRPs and CRP variabilities under all conditions (NF vs EF vs SF vs TF). *EF, SF, TF stands for elbow fatigue, shoulder fatigue and trunk fatigue condition respectively. ShHoAbd—ElFl stands for shoulder horizontal abduction—elbow flexion; TrFl—ShHoAbd stands for trunk flexion—shoulder horizontal abduction; TrRo—ShHoAbd stands for trunk rotation—shoulder horizontal abduction; TrLaFl—ShAbd stands for trunk lateral flexion—shoulder abduction; TrLaFl—ShHoAbd stands for trunk lateral flexion—shoulder horizontal abduction. * indicates that there was a main location effect. The values in the parentheses are the Wald Chi-Square value and the p values for joint angle x, y, z and 95% Confidence Interval for difference for the pairwise comparisons.
(DOCX)

S2 Table Maximum joint angles result under all conditions (NF vs EF vs SF vs TF). EF, SF, TF stands for elbow fatigue, shoulder fatigue and trunk fatigue condition respectively. Trunk x, y, z angles are trunk lateral flexion, rotation, flexion angles, respectively. Shoulder x, y, z angles are shoulder horizontal abduction, abduction, rotation angles, respectively, Elbow x, y, z angles are elbow flexion, abduction and rotation angles, respectively. * indicates that there was a main location effect. The values in the parenthesis are the Wald Chi-Square value and p values for joint angle x, y, z and 95% Confidence Interval for difference for the pairwise comparisons.
(DOCX)

S3 Table Minimum joint angles result under all conditions (NF vs EF vs SF vs TF). EF, SF, TF stands for elbow fatigue, shoulder fatigue and trunk fatigue condition respectively. Trunk x, y, z angles are trunk lateral flexion, rotation, flexion angles, respectively. Shoulder x, y, z angles are shoulder horizontal abduction, abduction, rotation angles, respectively, Elbow x, y, z angles are elbow flexion, abduction and rotation angles, respectively. * indicates that there was a main location effect. The values in the parenthesis are the Wald Chi-Square value and p values for joint angle x, y, z and 95% Confidence Interval for difference for the pairwise comparisons.
(DOCX)

S4 Table Joint angular variabilities result under all conditions (NF vs EF vs SF vs TF). EF, SF, TF stands for elbow fatigue, shoulder fatigue and trunk fatigue condition respectively. Trunk x, y, z angles are trunk lateral flexion, rotation, flexion angles, respectively. Shoulder x, y, z angles are shoulder horizontal abduction, abduction, rotation angles, respectively, Elbow x, y, z angles are elbow flexion, abduction and rotation angles, respectively. * indicates that there was a main location effect. The values in the parenthesis are the Wald Chi-Square value and p values for joint angle x, y, z and 95% Confidence Interval for difference for the pairwise comparisons.
(DOCX)
