# Supplementary material for: Effects of different fatigue locations on upper body kinematics and inter-joint coordination in a repetitive pointing task
Source: PLoS One. 2019 Dec 31;14(12):e0227247. doi: 10.1371/journal.pone.0227247 (PMC6938350; doi:10.1371/journal.pone.0227247)
Supplement: S1 Table — *EF, SF, TF stands for elbow fatigue, shoulder fatigue and trunk fatigue condition respectively. ShHoAbd—ElFl stands for shoulder horizontal abduction—elbow flexion; TrFl—ShHoAbd stands for trunk flexion—shoulder horizontal abduction; TrRo—ShHoAbd stands for trunk rotation—shoulder horizontal abduction; TrLaFl—ShAbd stands for trunk lateral flexion—shoulder abduction; TrLaFl—ShHoAbd stands for trunk lateral flexion—shoulder horizontal abduction. * indicates that there was a main location effect. The values in the parentheses are the Wald Chi-Square value and the p values for joint angle x, y, z and 95% Confidence Interval for difference for the pairwise comparisons. (DOCX) [file pone.0227247.s001.docx]

| **CRP&CRP variability** | | **NF** | **EF** | **SF** | **TF** |
| --- | --- | --- | --- | --- | --- |
| **ShPE - ElFl** | Mean CRP  (5.68, p=0.13) | SF: (-1.69, 4.56); p=0.55;  EF: (-5.11, 2.69); p=0.65;  TF: (-3.03, 3.65); p=0.86 | SF: (0.10, 5.20); p=0.17;  TF: (-0.62, 3.65); p=0.33;  NF: (-2.69, 5.11); p=0.65 | EF: (-5.20, -0.10); p=0.17;  TF: (-2.29, 0.03); p=0.17;  NF: (-4.56, 1.69); p=0.55 | SF: (-0.03, 2.29); p=0.17;  EF: (-3.65, 0.62); p=0.33;  NF: (-3.65, 3.03); p=0.86 |
|  | ****Variability***  ***(8.78, p=0.03)*** | SF: (-4.19, 2.08); p=0.93;  EF: (-1.57, 0.97); p=0.93;  TF: (-2.88, 3.03); p=0.96 | SF: (-3.60, 2.09); p=0.93;  TF: (-2.20, 2.95); p=0.93;  NF: (-0.97, 1.57); p=0.93 | EF: (-2.09, 3.60); p=0.93;  ****TF: (0.30, 1.96); p=0.04;***  NF: (-2.08, 4.19); p=0.93 | ****SF: (-1.96, -0.30); p=0.04;***  EF: (-2.95, 2.20); p=0.93;  NF: (-3.03, 2.88); p=0.96 |
| **TrFl - ShPE** | Mean CRP  (0.43, p=0.93) | SF: (-8.06, 8.00); p=0.99;  EF: (-6.08, 8.42); p=0.96;  TF: (-5.01, 8.28); p=0.96 | SF: (-8.06, 5.66); p=0.96;  TF: (-3.12, 4.05); p=0.96;  NF: (-8.42, 6.08); p=0.96 | EF: (-5.66, 8.06); p=0.96;  TF: (-4.66, 7.99); p=0.96;  NF: (-8.00, 8.06); p=0.99 | SF: (-7.99, 4.66); p=0.96;  EF: (-4.05, 3.12); p=0.96;  NF: (-8.28, 5.01); p=0.96 |
|  | Variability  (4.34, p=0.23) | SF: (-9.38, 9.89); p=0.96;  EF: (-1.72, 13.69); p=0.45;  TF: (-4.15, 10.91); p=0.67 | SF: (-13.52, 2.05); p=0.45;  TF: (-11.36, 6.15); p=0.67;  NF: (-13.69, 1.72); p=0.45 | EF: (-2.05, 13.52); p=0.45;  TF: (-5.79, 12.04); p=0.67;  NF: (-9.89, 9.38); p=0.96 | SF: (-12.04, 5.79); p=0.67;  EF: (-6.15, 11.36); p=0.67;  NF: (-10.91, 4.15); p=0.67 |
| **TrRo - ShPE** | ****Mean CRP***  ***(11.06, p=0.01)*** | SF: (-0.85, 4.66); p=0.26;  EF: (-2.88, 3.88); p=0.77;  ****TF: (-6.52, 0.82); p=0.03*** | SF: (-2.25, 5.06); p=0.54;  TF: (-8.04, 0.31); p=0.06;  NF: (-3.88, 2.88); p=0.77 | EF: (-5.06, 2.25); p=0.54;  ****TF: (-8.96, -2.20); p<0.01;***  NF: (-4.66, 0.85); p=0.26 | ****SF: (2.20, 8.96); p<0.01;***  EF: (0.31, 8.04); p=0.06;  ****NF: (0.82, 6.52); p=0.03*** |
|  | Variability  (1.10, p=0.77) | SF: (-1.98, 4.76); p=0.89;  EF: (-1.58, 4.28); p=0.89;  TF: (-1.80, 3.71); p=0.89 | SF: (-3.44, 3.52); p=0.98;  TF: (-3.42, 2.63); p=0.96;  NF: (-4.28, 1.58); p=0.89 | EF: (-3.52, 3.44); p=0.98;  TF: (-2.05, 1.17); p=0.89;  NF: (-4.76, 1.98); p=0.89 | SF: (-1.17, 2.05); p=0.89;  EF: (-2.63, 3.42); p=0.96;  NF: (-3.71, 1.80); p=0.89 |
| **TrLaFl - ShEl** | ****Mean CRP***  ***(9.66, p=0.02)*** | SF: (-6.72, 9.53); p=0.74;  EF: (-11.53, 5.50); p=0.59;  TF: (-18.39, -1.66);p=0.06 | SF: (-4.93, 13.78); p=0.53;  TF: (-14.36, 0.33); p=0.12;  NF: (-5.50, 11.53); p=0.59 | EF: (-13.78, 4.93); p=0.53;  ****TF: (-19.48,-3.39);p=0.03;***  NF: (-9.53, 6.72); p=0.74 | ****SF: (3.39, 19.48); p=0.03;***  EF: (-0.33, 14.36); p=0.12;  NF: (1.66, 18.39); p=0.06 |
|  | Variability  (3.46, p=0.33) | SF: (-10.32, 15.42); p=0.80  EF: (-11.84, 5.44); p=0.70  TF: (-12.68, 4.76); p=0.70 | SF: (-2.33, 13.83); p=0.56;  TF: (-6.53, 5.01); p=0.80;  NF: (-5.44, 11.84); p=0.70 | EF: (-13.83, 2.33); p=0.56;  TF: (-16.16, 3.14); p=0.56;  NF: (-15.42, 10.32); p=0.80 | SF: (-3.14, 16.16); p=0.56;  EF: (-5.01, 6.53); p=0.80;  NF: (-4.76, 12.68); p=0.70 |
| **TrLaFl - ShPE** | Mean CRP  (5.96, p=0.11) | SF: (-7.77, 5.55); p=0.74;  EF: (-9.61, 4.60); p=0.73;  TF: (-14.92, 0.55); p=0.18 | SF: (-6.02, 8.81); p=0.74;  TF: (-9.15, -0.20); p=0.18;  NF: (-4.60, 9.61); p=0.73 | EF: (-8.81, 6.02); p=0.74;  TF: (-13.08, 0.94); p=0.18;  NF: (-5.55, 7.77); p=0.74 | SF: (-0.94, 13.08); p=0.18;  EF: (0.20, 9.15); p=0.18***;***  NF: (-0.55, 14.92); p=0.18 |
|  | Variability  (5.31, p=0.15) | SF: (-5.13, 14.69); p=0.58;  EF: (-10.68, 6.05); p=0.61;  TF: (-12.76, 4.95); p=0.58 | SF: (0.03, 14.16); p=0.15;  TF: (-7.66, 4.48); p=0.61;  NF: (-6.05, 10.68); p=0.61 | EF: (-14.16, -0.03); p=0.15;  TF: (-16.62, 0.75); p=0.15;  NF: (-14.69, 5.13); p=0.58 | SF: (0.75, 16.62); p=0.15;  EF: (-4.48, 7.66); p=0.61;  NF: (-4.95, 12.76); p=0.58 |

**Table 1. Mean CRPs and CRP Variabilities under All Conditions (NF vs EF vs SF vs TF)**

EF, SF, TF stands for elbow fatigue, shoulder fatigue and trunk fatigue condition respectively. ShPE-ElFl, TrFl-ShPE, TrRo-ShPE, TrLaFl-ShEl, TrLaFl-ShPE indicate CRP between shoulder plane of elevation and elbow flexion, CRP between trunk flexion and shoulder plane of elevation, CRP between trunk rotation and shoulder plane of elevation, CRP between trunk lateral flexion and shoulder elevation, CRP between trunk lateral flexion and shoulder plane of elevation, respectively. * indicates that there was a main location effect. The values in the parenthesis are the Wald Chi-Square value and the corrected p values for joint angle x, y, z and 95% Confidence Interval for difference for the pairwise comparisons.
