# Supplementary material for: Effects of different fatigue locations on upper body kinematics and inter-joint coordination in a repetitive pointing task
Source: PLoS One. 2019 Dec 31;14(12):e0227247. doi: 10.1371/journal.pone.0227247 (PMC6938350; doi:10.1371/journal.pone.0227247)
Supplement: S2 Table — EF, SF, TF stands for elbow fatigue, shoulder fatigue and trunk fatigue condition respectively. Trunk x, y, z angles are trunk lateral flexion, rotation, flexion angles, respectively. Shoulder x, y, z angles are shoulder horizontal abduction, abduction, rotation angles, respectively, Elbow x, y, z angles are elbow flexion, abduction and rotation angles, respectively. * indicates that there was a main location effect. The values in the parenthesis are the Wald Chi-Square value and p values for joint angle x, y, z and 95% Confidence Interval for difference for the pairwise comparisons. (DOCX) [file pone.0227247.s002.docx]

**Table 2. Maximum joint angles result under all conditions (NF vs EF vs SF vs TF)**

| **Maximum Angles** | | **NF** | **EF** | **SF** | **TF** |
| --- | --- | --- | --- | --- | --- |
| **Trunk** | ****X***  ***(15.88, p<0.01)*** | ****SF: (-3.61, -0.42); p=0.03;***  ****EF: (0.13, 4.88); p=0.04;***  TF: (-1.79, 1.06); p=0.61 | ****SF: (-6.88, -2.16); p<0.01;***  ****TF: (-5.08, -0.67); p=0.03;***  ****NF: (-4.88, -0.13); p=0.04*** | ****EF: (2.16, 6.88); p<0.01;***  ****TF: (0.08, 3.22); p=0.04;***  ****NF: (0.42, 3.61); p=0.03*** | ****SF: (-3.22, -0.08); p=0.04;***  ****EF: (0.67, 5.08); p=0.03;***  NF: (-1.06, 1.79); p=0.61 |
|  | ****Y***  ***(35.62, p<0.01)*** | ****SF: (2.03, 4.48); p<0.01;***  EF: (-0.19, 1.48); p=0.20;  TF: (-1.09, 0.79); p=0.76 | ****SF: (1.54, 3.68); p<0.01;***  TF: (-1.96, 0.37); p=0.22;  NF: (-1.48, 0.19); p=0.19 | ****EF: (-3.68, -1.54); p<0.01;***  ****TF: (-4.63, -2.17); p<0.01;***  ****NF: (-4.48, -2.03); p<0.01*** | ****SF: (2.17, 4.63); p<0.01;***  EF: (-0.37, 1.96); p=0.22;  NF: (-0.79, 1.09); p=0.76 |
|  | Z  (4.63, p=0.20) | SF: (-1.34, 0.46); p=0.59;  EF: (-0.92, 1.02); p=0.92;  TF: (-0.72, 1.66); p=0.59 | SF: (-1.33, 0.35); p=0.59;  TF: (-0.78, 1.62); p=0.59;  NF: (-1.02, 0.92); p=0.92 | EF: (-0.35, 1.33); p=0.59;  TF: (-0.03, 1.85); p=0.34;  NF: (-0.46, 1.34); p=0.59 | SF: (-1.85, 0.03); p=0.34;  EF: (-1.62, 0.78); p=0.59;  NF: (-1.66, 0.72); p=0.59 |
| **Shoulder** | ****X***  ***(14.47, p<0.01)*** | SF: (0.36, 5.86); p=0.05;  EF: (-3.22, 2.59); p=0.83;  TF: (-4.30, 0.68); p=0.19 | ****SF: (1.07, 5.78); p=0.01;***  TF: (-3.49, 0.51); p=0.19;  NF: (-2.59, 3.22); p=0.83 | ****EF: (-5.78, -1.07); p=0.01;***  ****TF: (-7.47, -2.37); p<0.01;***  NF: (-5.86, -0.36); p=0.05 | ****SF: (2.37, 7.47); p<0.01;***  EF: (-0.51, 3.49); p=0.19;  NF: (-0.68, 4.30); p=0.19 |
|  | ****Y***  ***(33.26, p<0.01)*** | ****SF: (-7.49, -1.84); p<0.01;***  ****EF: (2.82, 6.21); p<0.01;***  ****TF: (0.43, 3.97); p=0.02*** | ****SF: (-12.70, -5.64); p<0.01***  ****TF: (-3.98, -0.65); p<0.01;***  ****NF: (-6.21, -2.82); p<0.01*** | ****EF: (5.64, 12.70); p<0.01;***  ****TF: (3.91, 9.81); p<0.01;***  ****NF: (1.84, 7.49); p<0.01*** | ****SF: (-9.81, -3.91); p<0.01;***  ****EF: (0.65, 3.98); p=0.01;***  ****NF: (-3.97, -0.43); p=0.02*** |
|  | Z  (1.04, p=0.79) | SF: (-3.39, 2.37); p=0.91;  EF: (-2.92, 1.40); p=0.91;  TF: (-2.90, 3.17); p=0.93 | SF: (-1.36, 1.86); p=0.91;  TF: (-1.38, 3.16); p=0.91;  NF: (-1.40, 2.92); p=0.91 | EF: (-1.86, 1.36); p=0.91;  TF: (-1.69, 2.98); p=0.91;  NF: (-2.73, 3.39); p=0.91 | SF: (-2.98, 1.69); p=0.91;  EF: (-3.16, 1.38); p=0.91;  NF: (-3.17, 2.90); p=0.93 |
| **Elbow** | X  (6.84, p=0.08) | SF: (-4.78, -0.51); p=0.09;  EF: (-2.86, 0.45); p=0.32;  TF: (-3.50, 1.45); p=0.50 | SF: (-3.70, 0.83); p=0.32;  TF: (-2.39, 2.75); p=0.89;  NF: (-0.45, 2.86); p=0.32 | EF: (-0.83, 3.70); p=0.32;  TF: (-0.64, 3.87); p=0.32;  NF: (-1.45, 3.50); p=0.50 | SF: (-3.87, 0.64); p=0.32;  EF: (-2.75, 2.39); p=0.89;  NF: (-1.45, 3.50); p=0.50 |
|  | ****Y***  ***(16.59, p<0.01)*** | SF: (-1.75, 0.36); p=0.29;  ****EF: (-2.98, -0.47); p=0.02;***  TF: (-1.89, 1.03); p=0.57 | ****SF: (0.17, 1.88); p=0.04;***  ****TF: (0.46, 2.13); p=0.01;***  ****NF: (0.47, 2.98); p=0.02*** | ****EF: (-1.88, -0.17); p=0.04;***  TF: (-0.52, 1.06); p=0.57;  NF: (-0.36, 1.75); p=0.29 | SF: (-1.06, 0.52); p=0.57;  ****EF: (-2.13, -0.46); p=0.01;***  NF: (-1.03, 1.89); p=0.57 |
|  | ****Z***  ***(13.09, p=0.01)*** | SF: (-6.09, 4.81); p=0.88;  ****EF: (2.59, 13.87); p=0.01;***  TF: (-5.96, 5.12); p=0.88 | ****SF: (-14.43, -3.30); p=0.01***  ****TF: (-13.77, -3.52); p=0.01***  ****NF:(-13.87, -2.59); p=0.01*** | ****EF: (3.30, 14.43); p=0.01;***  TF: (-2.25, 2.69); p=0.88;  NF: (-4.81, 6.09); p=0.88 | SF: (-2.25, 2.69); p=0.88;  ****EF: (3.52, 13.77); p=0.01;***  NF: (-5.12, 5.96); p=0.88 |

EF, SF, TF stands for elbow fatigue, shoulder fatigue and trunk fatigue condition respectively. Trunk x, y, z angles are trunk lateral flexion, rotation, flexion angles, respectively. Shoulder x, y, z angles are shoulder plane of elevation, elevation, rotation angles, respectively, Elbow x, y, z angles are elbow flexion, abduction and rotation angles, respectively. * indicates that there was a main location effect. The values in the parenthesis are the Wald Chi-Square value and the corrected p values for joint angle x, y, z and 95% Confidence Interval for difference for the pairwise comparisons.
