# Supplementary material for: Effects of different fatigue locations on upper body kinematics and inter-joint coordination in a repetitive pointing task
Source: PLoS One. 2019 Dec 31;14(12):e0227247. doi: 10.1371/journal.pone.0227247 (PMC6938350; doi:10.1371/journal.pone.0227247)
Supplement: S3 Table — EF, SF, TF stands for elbow fatigue, shoulder fatigue and trunk fatigue condition respectively. Trunk x, y, z angles are trunk lateral flexion, rotation, flexion angles, respectively. Shoulder x, y, z angles are shoulder horizontal abduction, abduction, rotation angles, respectively, Elbow x, y, z angles are elbow flexion, abduction and rotation angles, respectively. * indicates that there was a main location effect. The values in the parenthesis are the Wald Chi-Square value and p values for joint angle x, y, z and 95% Confidence Interval for difference for the pairwise comparisons. (DOCX) [file pone.0227247.s003.docx]

**Table 3. Minimum joint angles result under all conditions (NF vs EF vs SF vs TF)**

| **Minimum Angles** | | **NF** | **EF** | **SF** | **TF** |
| --- | --- | --- | --- | --- | --- |
| **Trunk** | ****X***  ***(13.85, p<0.01)*** | SF: (-3.41, 0.03); p=0.07;  ****EF: (0.40, 5.06); p=0.04;***  TF: (-1.48, 1.36); p=0.94 | ****SF: (-6.78, -2.06); p<0.01;***  ****TF: (-4.97, -0.60); p=0.03;***  ****NF: (-5.06, -0.40); p=0.04*** | ***EF: (2.06, 6.78); p<0.01;***  TF: (0.02, 3.24); p=0.06;  NF: (-0.03, 3.41); p=0.06 | SF: (-3.24, -0.02); p=0.06;  ****EF: (0.60, 4.97); p=0.04;***  NF: (-1.36, 1.48); p=0.94 |
|  | ****Y***  ***(37.24, p<0.01)*** | ****SF: (2.13, 5.02); p<0.01;***  EF: (-0.31, 1.52); p=0.23;  TF: (-1.32, 0.81); p=0.64 | ****SF: (1.84, 4.11); p<0.01;***  TF: (-2.04, 0.32); p=0.23;  NF: (-1.52, 0.31); p=0.23 | ****EF: (-4.11, -1.84); p<0.01;***  ****TF: (-5.18, -2.149); p<0.01***  ****NF: (-5.02, -2.13); p<0.01*** | ****SF: (2.49, 5.18); p<0.01;***  EF: (-0.32, 2.04); p=0.23;  NF: (-0.81, 1.32); p=0.64 |
|  | Z  (3.21, p=0.36) | SF: (-0.60, 1.18); p=0.71;  EF: (-0.08, 1.58); p=0.45;  TF: (-0.86, 1.72); p=0.71 | SF: (-1.39, 0.46); p=0.71;  TF: (-1.51, 0.86); p=0.71;  NF: (-1.58, 0.08); p=0.45 | EF: (-0.46, 1.39); p=0.71;  TF: (-0.80, 1.08); p=0.77;  NF: (-1.18, 0.60); p=0.71 | SF: (-1.08, 0.80); p=0.77;  EF: (-0.86, 1.51); p=0.71;  NF: (-1.72, 0.86); p=0.71 |
| **Shoulder** | ****X***  ***(9.77, p=0.02)*** | SF: (0.33, 5.15); p=0.06;  EF: (-2.19, 2.53); p=0.96;  TF: (-2.27, 2.52); p=0.96 | SF: (0.27, 4.87); p=0.06;  TF: (-2.07, 1.98); p=0.96;  NF: (-2.53, 2.19); p=0.96 | EF: (-4.87, -0.27); p=0.06;  ****TF: (-4.41, -0.82); p<0.01;***  NF: (-5.15, -0.33); p=0.06 | ****SF: (0.82, 4.41); p<0.01;***  EF: (-1.98, 2.07); p=0.96;  NF: (-2.52, 2.27); p=0.96 |
|  | ****Y***  ***(56.97, p<0.01)*** | ****SF: (-8.46, -2.44); p<0.01;***  ****EF: (2.32, 5.10); p<0.01;***  ****TF: (0.52, 3.78); p=0.01*** | ****SF: (-12.11, -6.20); p<0.01***  ****TF: (-2.51, -0.61); p<0.01;***  ****NF: (-5.10, -2.32); p<0.01*** | ****EF: (6.20, 12.11); p<0.01;***  ****TF: (4.71, 10.48); p<0.01;***  ****NF: (2.44, 8.46); p<0.01*** | ****SF: (-10.48, -4.71); p<0.01***  ****EF: (0.61, 2.51); p<0.01;***  ****NF: (-3.78, -0.52); p=0.01*** |
|  | Z  (3.03, p=0.39) | SF: (-1.39, 5.36); p=0.50;  EF: (-4.30, 2.43); p=0.70;  TF: (-4.19, 3.97); p=0.96 | SF: (-0.44, 6.28); p=0.50;  TF: (-1.78, 3.43); p=0.70;  NF: (-2.43, 4.30); p=0.70 | EF: (-6.28, 0.44); p=0.50;  TF: (-5.46, 1.28); p=0.50;  NF: (-5.36, 1.39); p=0.50 | SF: (-1.28, 5.46); p=0.50;  EF: (-3.43, 1.78); p=0.70;  NF: (-3.97, 4.19); p=0.96 |
| **Elbow** | ****X***  ***(85.30, p<0.01)*** | SF: (-4.57, 0.08); p=0.09;  EF: (-4.57, 0.08); p=0.29;  ****TF: (-1.73, 3.78); p<0.01*** | ****SF: (-5.46, -1.43); p<0.01;***  TF: (-0.83, 3.95); p=0.24;  NF: (-0.08, 4.57); p=0.29 | ****EF: (1.43, 5.46); p<0.01;***  ****TF: (-2.96, 7.04); p<0.01;***  NF: (-0.08, 4.57); p=0.09 | ****SF: (-7.04, 2.96); p<0.01;***  EF: (-3.95, 0.83); p=0.24;  ****NF: (-3.78, 1.73); p<0.01*** |
|  | Y  (1.45, p=0.69) | SF: (-0.83, 0.80); p=0.97;  EF: (-1.20, -0.43); p=0.85;  TF: (-0.42, 0.70); p=0.85 | SF: (0.59, 1.33); p=0.85;  TF: (-0.33, 1.38); p=0.85;  NF: (-0.43, 1.20); p=0.85 | EF: (-1.33, 0.59); p=0.85;  TF: (-0.66, 0.98); p=0.85;  NF: (-0.80, 0.83); p=0.97 | SF: (-0.98, 0.66); p=0.85;  EF: (-1.38, 0.33); p=0.85;  NF: (-0.70, 0.42); p=0.85 |
|  | ****Z***  ***(18.09, p<0.01)*** | SF: (-4.82, 9.05); p=0.66;  ****EF: (4.30, 13.53); p<0.01;***  TF: (-4.90, 5.60); p=0.90 | SF: (-14.79, 1.19); p=0.20;  ****TF: (-14.10, -3.04); p=0.01***  ****NF: (-13.53, -4.30);p<0.01*** | EF: (-1.19, 14.79); p=0.20;  TF: (-7.61, 4.07); p=0.66;  NF: (-9.05, 4.82); p=0.66 | SF: (-4.07, 7.61); p=0.66;  ****EF: (3.04, 14.10); p=0.01;***  NF: (-5.60, 4.90); p=0.90 |

EF, SF, TF stands for elbow fatigue, shoulder fatigue and trunk fatigue condition respectively. Trunk x, y, z angles are trunk lateral flexion, rotation, flexion angles, respectively. Shoulder x, y, z angles are shoulder plane of elevation, elevation, rotation angles, respectively, Elbow x, y, z angles are elbow flexion, abduction and rotation angles, respectively. * indicates that there was a main location effect. The values in the parenthesis are the Wald Chi-Square value and the corrected p values for joint angle x, y, z and 95% Confidence Interval for difference for the pairwise comparisons.
