# Supplementary material for: Effects of different fatigue locations on upper body kinematics and inter-joint coordination in a repetitive pointing task
Source: PLoS One. 2019 Dec 31;14(12):e0227247. doi: 10.1371/journal.pone.0227247 (PMC6938350; doi:10.1371/journal.pone.0227247)
Supplement: S4 Table — EF, SF, TF stands for elbow fatigue, shoulder fatigue and trunk fatigue condition respectively. Trunk x, y, z angles are trunk lateral flexion, rotation, flexion angles, respectively. Shoulder x, y, z angles are shoulder horizontal abduction, abduction, rotation angles, respectively, Elbow x, y, z angles are elbow flexion, abduction and rotation angles, respectively. * indicates that there was a main location effect. The values in the parenthesis are the Wald Chi-Square value and p values for joint angle x, y, z and 95% Confidence Interval for difference for the pairwise comparisons. (DOCX) [file pone.0227247.s004.docx]

**Table 4. Joint angular variabilities result under all conditions (NF vs EF vs SF vs TF)**

| **Angular variability** | | **NF** | **EF** | **SF** | **TF** |
| --- | --- | --- | --- | --- | --- |
| **Trunk** | ****X***  ***(18.83, p<0.01)*** | SF: (-0.21, -0.01); p=0.08;  EF: (-0.16, 0.00); p=0.08;  ****TF: (-0.16, 0.06); p<0.01*** | SF: (-0.13, 0.07); p=0.71;  TF: (-0.08, 0.03); p=0.53;  NF: (0.00, 0.16); p=0.08 | EF: (-0.07, 0.13); p=0.71;  TF: (-0.08, 0.08); p=0.98;  NF: (0.01, 0.21); p=0.08 | SF: (0.08, 0.08); p=0.98;  EF: (-0.03, 0.08); p=0.53;  ****NF: (0.06, 0.16); p<0.01*** |
|  | ****Y***  ***(13.13, p<0.01)*** | SF: (-0.24, 0.03); p=0.27;  EF: (-0.09, 0.13); p=0.70;  TF: (-0.06, 0.15); p=0.47 | ****SF: (-0.19, 0.05); p<0.01;***  TF: (-0.03, 0.08); p=0.47;  NF: (-0.13, 0.09); p=0.70 | ****EF: (0.05, 0.19); p<0.01;***  ****TF: (0.06, 0.24); p<0.01;***  NF: (-0.03, 0.24); p=0.27 | ****SF: (-0.24, -0.06); p<0.01;***  EF: (-0.08, 0.03); p=0.47;  NF: (-0.15, 0.06); p=0.47 |
|  | Z  (7.17, p=0.07) | SF: (-0.54, 0.04); p=0.17;  EF: (-0.49, 0.05); p=0.17;  TF: (-0.26, 0.31); p=0.87 | SF: (-0.25, 0.19); p=0.87;  TF: (0.02, 0.46); p=0.11;  NF: (-0.05, 0.49); p=0.17 | EF: (-0.19, 0.25); p=0.87;  TF: (0.02, 0.52); p=0.11;  NF: (-0.04, 0.54); p=0.17 | SF: (-0.52, -0.02); p=0.11;  EF: (-0.46, -0.02); p=0.11;  NF: (-0.31, 0.26); p=0.87 |
| **Shoulder** | ****X***  ***(8.15, p=0.04)*** | SF: (-0.63, 0.90); p=0.82;  EF: (-0.95, 0.76); p=0.82;  TF: (-1.21, -0.13); p=0.10 | SF: (-0.88, 1.35); p=0.82;  TF: (-1.37, 0.22); p=0.32;  NF: (-0.76, 0.95); p=0.82 | EF: (-1.35, 0.88); p=0.82;  TF: (-1.60, -0.01); p=0.14;  NF: (-0.90, 0.63); p=0.82 | SF: (0.01, 1.60); p=0.14;  EF: (-0.22, 1.37); p=0.32;  NF: (0.13, 1.21); p=0.10 |
|  | Y  (3.16, p=0.37) | SF: (-0.07, 0.72); p=0.39;  EF: (-0.25, 0.88); p=0.40;  TF: (-0.35, 0.36); p=0.99 | SF: (-0.46, 0.47); p=0.99;  TF: (-0.80, 0.16); p=0.39;  NF: (-0.88, 0.25); p=0.40 | EF: (-0.47, 0.46); p=0.99;  TF: (-0.75, 0.11); p=0.39;  NF: (-0.72, 0.07); p=0.39 | SF: (-0.11, 0.75); p=0.39;  EF: (-0.16, 0.80); p=0.40;  NF: (-0.36, 0.35); p=0.99 |
|  | Z  (4.56, p=0.21) | SF: (-1.97, 0.10); p=0.16;  EF: (-1.00, 1.06); p=0.96;  TF: (-0.98, 0.93); p=0.96 | SF: (-1.96, 0.03); p=0.16;  TF: (-0.46, 0.35); p=0.96;  NF: (-1.06, 1.00); p=0.96 | EF: (-0.03, 1.96); p=0.16;  TF: (-0.05, 1.87); p=0.16;  NF: (-0.10, 1.97); p=0.18 | SF: (-1.87, 0.46); p=0.16;  EF: (-0.35, 0.46); p=0.96;  NF: (-0.93, 0.98); p=0.96 |
| **Elbow** | ****X***  ***(16.55, p<0.01)*** | SF: (-0.99, 1.03); p=0.97;  EF: (-1.68, 0.05); p=0.10;  ****TF: (-2.15, 0.51); p<0.01*** | SF: (-0.04, 1.69); p=0.10;  TF: (-1.20, 0.16); p=0.16;  NF: (-0.05, 1.68); p=0.10 | EF: (-1.69, 0.04); p=0.10;  ****TF: (-2.17, -0.53); p<0.01***  NF: (-1.03, 0.99); p=0.97 | ****SF: (0.53, 2.17); p<0.01;***  EF: (-0.16, 1.20); p=0.16;  ****NF: (0.51, 2.15); p<0.01*** |
|  | Y  (5.34 p=0.15) | SF: (-0.73, 0.16); p=0.57;  EF: (-0.94, -0.10); p=0.57;  TF: (-0.71, 0.30); p=0.60 | SF: (-0.34, 0.60); p=0.60;  TF: (0.18, 0.60); p=0.57;  NF: (0.10, 0.94); p=0.57 | EF: (-0.60, 0.34); p=0.60;  TF: (-0.16, 0.33); p=0.60;  NF: (-0.16, 0.73); p=0.57 | SF: (-0.16, 0.33); p=0.60;  EF: (-0.60, 0.18); p=0.57;  NF: (-0.30, 0.71); p=0.60 |
|  | Z  (1.47, p=0.69) | SF: (-2.92, 0.77); p=0.81;  EF: (-1.59, 1.28); p=0.83;  TF: (-1.77, 1.15); p=0.81 | SF: (-2.88, 1.04); p=0.81;  TF: (-0.74, 0.44); p=0.81;  NF: (-1.28, 1.59); p=0.83 | EF: (-1.04, 2.88); p=0.81;  TF: (-1.16, 2.69); p=0.81;  NF: (-0.77, 2.92); p=0.81 | SF: (-2.69, 1.16); p=0.81;  EF: (-0.44, 0.74); p=0.81;  NF: (-1.15, 1.77); p=0.81 |

EF, SF, TF stands for elbow fatigue, shoulder fatigue and trunk fatigue condition respectively. Trunk x, y, z angles are trunk lateral flexion, rotation, flexion angles, respectively. Shoulder x, y, z angles are shoulder plane of elevation, elevation, rotation angles, respectively, Elbow x, y, z angles are elbow flexion, abduction and rotation angles, respectively. * indicates that there was a main location effect. The values in the parenthesis are the Wald Chi-Square value and the corrected p values for joint angle x, y, z and 95% Confidence Interval for difference for the pairwise comparisons.
